# A self-powered wireless motion sensor based on a high-surface area reverse electrowetting-on-dielectric energy harvester

**DOI:** 10.1038/s41598-022-07631-4

**Published:** 2022-03-08

**Authors:** Nishat T. Tasneem, Dipon K. Biswas, Pashupati R. Adhikari, Avinash Gunti, Adnan B. Patwary, Russell C. Reid, Ifana Mahbub

**Affiliations:** 1 Department of Electrical Engineering, University of North Texas, Denton, TX 76201 USA; 2 Department of Mechanical Engineering, University of North Texas, Denton, TX 76201 USA; 3 Department of Engineering, Dixie State University, St. George, UT 84770 USA

**Keywords:** Electrical and electronic engineering, Mechanical engineering

## Abstract

This paper presents a motion-sensing device with the capability of harvesting energy from low-frequency motion activities. Based on the high surface area reverse electrowetting-on-dielectric (REWOD) energy harvesting technique, mechanical modulation of the liquid generates an AC signal, which is modeled analytically and implemented in Matlab and COMSOL. A constant DC voltage is produced by using a rectifier and a DC–DC converter to power up the motion-sensing read-out circuit. A charge amplifier converts the generated charge into a proportional output voltage, which is transmitted wirelessly to a remote receiver. The harvested DC voltage after the rectifier and DC–DC converter is found to be 3.3 V, having a measured power conversion efficiency (PCE) of the rectifier as high as 40.26% at 5 Hz frequency. The energy harvester demonstrates a linear relationship between the frequency of motion and the generated output power, making it highly suitable as a self-powered wearable motion sensor.

## Introduction

Advanced research on wireless wearable bioelectronic devices have been extensively proliferated while broadening the scope of health monitoring systems^1–5^. Wireless wearable systems provide the advantage of utilizing a noninvasive method of deriving physical activities and sending those to a remote user. Typical wearable devices detect vital health signs such as pulse waveform^6^, heart rate^7^, blood oxygen saturation^8^, respiration rate^9^, sweat analyte^10^ etc., and incorporate these into everyday lives. Motion is such a desirable health feature for the early detection of any muscular disorders and internal injuries^11,12^. Motion-related information also enhances fitness and health analytics for players by tracking motion continuously^13^. In recent decades, human physical activity sensors have seen exceptional growth in transmitting the extracted information for daily monitoring. However, powering these devices is a crucial challenge for long-term monitoring of wearable motion sensors^14^.

Wearable sensors mostly depend on battery power for powering the associated electronic circuitry for acquiring and transmitting the motion data. However, they have limitations such as charging and replacement, skin irritation, to name a few. As an alternative approach, energy is harvested from human motion to power the devices^10,14,15^. Several mechanisms are widely used in harvesting energy from motion, such as Lenz’s law-based electromagnetics, piezoelectric materials-based piezoelectricity, variable capacitance-based electrostatics, triboelectric nanogenerators (TENGs), reverse electrowetting-on-dielectric (REWOD) energy harvester, etc.^10,16–20^. Although the other harvesters provide attractive strategies to power the wearable devices, they mainly operate during intensive physical activities. Long-term stability also becomes a concern due to the contact surface degradation resulting from the interface friction^21^. Luo et al. proposed a non-resonant electromagnetic energy harvester with a rotation structure for detecting human/vehicle motion^22^. Although it reports a high energy conversion efficiency, it requires a sturdy drive force to convert a pulse-based motion to a high-speed rotation (140 rps)^23^. Here, we propose a self-powered motion sensor based on REWOD energy harvester for monitoring human physical activities, such as walking, running, etc. REWOD harvests energy by utilizing the electrical double-layer (EDL) capacitance mechanism. In a REWOD-based energy harvester, electrical power is generated from contact surface area modulation of the conductive liquid due to external mechanical force^24,25^. Since REWOD can generate comparatively high output power in a low-force, and low-frequency (<10 Hz) operation while covering a wide range of mechanical force, that is why REWOD is highly suitable for self-powered wearable motion-sensing applications.

Most of the REWOD energy harvesting techniques have used external bias voltages to start up the generated power to the electronic load. In a self-powered wearable motion sensor, it is undesirable to use batteries or external power sources to start up the process, thus a bias-free approach to generate electricity is used in this work^26^. Due to the increasing demand for miniaturized wearable sensors, the footprint of the sensor needs to be significantly decreased. Our prior works explored the planar surface area-based REWOD sensor^26–28^, while this work includes a porous high surface area-based REWOD sensor^29^. Our previous works shows the detailed performance comparison of the porous vs. planar REWOD and demonstrate how that enhances the power density^29^. In order to study the effects of pores in the generated power density, an analytical model is developed in this paper considering the important parameters, such as the dielectric material and its thickness, pore diameter, pore depth, depth-to-diameter aspect ratio, surface charge density, modulation frequency, and applied mechanical pressure. This work is a complete integrated system illustrating the modeling and the electrical circuitry of the self-powered motion sensor. The analytical model is implemented using MATLAB and COMSOL Multiphysics-based modeling and compared with the measured power densities and generated voltages to validate the performance.

In this work, a self-powered motion sensor is developed by integrating a commercial-off-the-shelf (COTS) components-based electronic interface with the REWOD transducer. The transducer produces an AC signal while working as an electrical energy harvester. Thus, an AC-to-DC signal converter/rectifier is required to provide a constant DC voltage to the signal conditioning circuitry. Only the rectifier cannot supply enough power to the system. The rectifier-generated energy is stored in a battery/supercapacitor, which is generally followed by a DC–DC converter or a low drop-out (LDO) voltage regulator. Since the generated signal from the REWOD is a very low-frequency signal, large capacitors are required to store the charge, which is difficult to achieve on-chip because of the area constraints. Thus, this work involves commercial components for the energy harvesting circuitry. This paper includes a bridge rectifier and a DC–DC boost converter connected to the harvester output to harvest sufficient energy to the signal conditioning circuitry.

Typical human body movements or physical activities can be monitored using two methods: vision-based activity recognition and inertial measurement units (IMUs)^30,31^. Although vision-based systems acquire motion-related information with high accuracy, they become unsuitable for activities in places where it is difficult to put video cameras. Thus, recently, IMU-based motion sensors have been studied extensively, which include accelerometers, gyroscopes, etc. as the motion sensors^32^. However, these systems become bulky when integrated with power sources. We propose REWOD-based motion sensors for monitoring daily physical activities such as running and walking, the frequency of which ranges from 0.25 to 5 Hz^33,34^. Since REWOD uses the low-frequency mechanical modulation to harvest energy, we can avail of this feature by utilizing it as the motion sensor. The basic idea is to translate human motion into electrical signals. In this work, an analog-to-digital converter (ADC) and a transmitter are included to send the output signals wirelessly to the remote receiver.

Due to the mechanical modulation, electrical charge is generated in the porous-REWOD electrode, which is considered as the motion data for a certain modulation/motion frequency. The generated charge is transformed into proportional electrical voltage with the help of a charge amplifier^35^. A low-pass filter is also designed by using an RC-feedback network. Prior works have presented both the COTS and application-specific integrated circuit (ASIC) implementation of charge amplifiers for wearable motion sensors, but most of the integrated electronics (IE) are designed for intensive physical activities^36,37^. In this work, we are using a COTS-based motion sensor system. The motion data are transmitted wirelessly via the CC430F5137 microcontroller (MCU) and transceiver IC (Integrated Circuit). The data are further processed in the receiver end. The received digitized data are transformed to the analog domain for further processing. The baseline wanders and high-frequency noise components are also eliminated from the received data in Matlab.

The novelty of this paper incorporates the modeling of the high surface area porous REWOD sensor and the low-power electronic circuit interfaces. The contributions of this work are as follows: (1) Generating high power output from low-frequency (1–5 Hz) motion activities without any external bias voltage, (2) Analytical and multi-physics based models of the porous REWOD electrode implemented in MATLAB and COMSOL, (3) Design of COTS-based energy harvesting circuit achieving a high power conversion efficiency, (4) Design of COTS-based motion-sensing low-power read-out circuit with wireless transmission capabilities, and (5) experimental validation of the REWOD to validate the models and the sensor read-out and energy harvesting circuitries.

**Figure 1:**
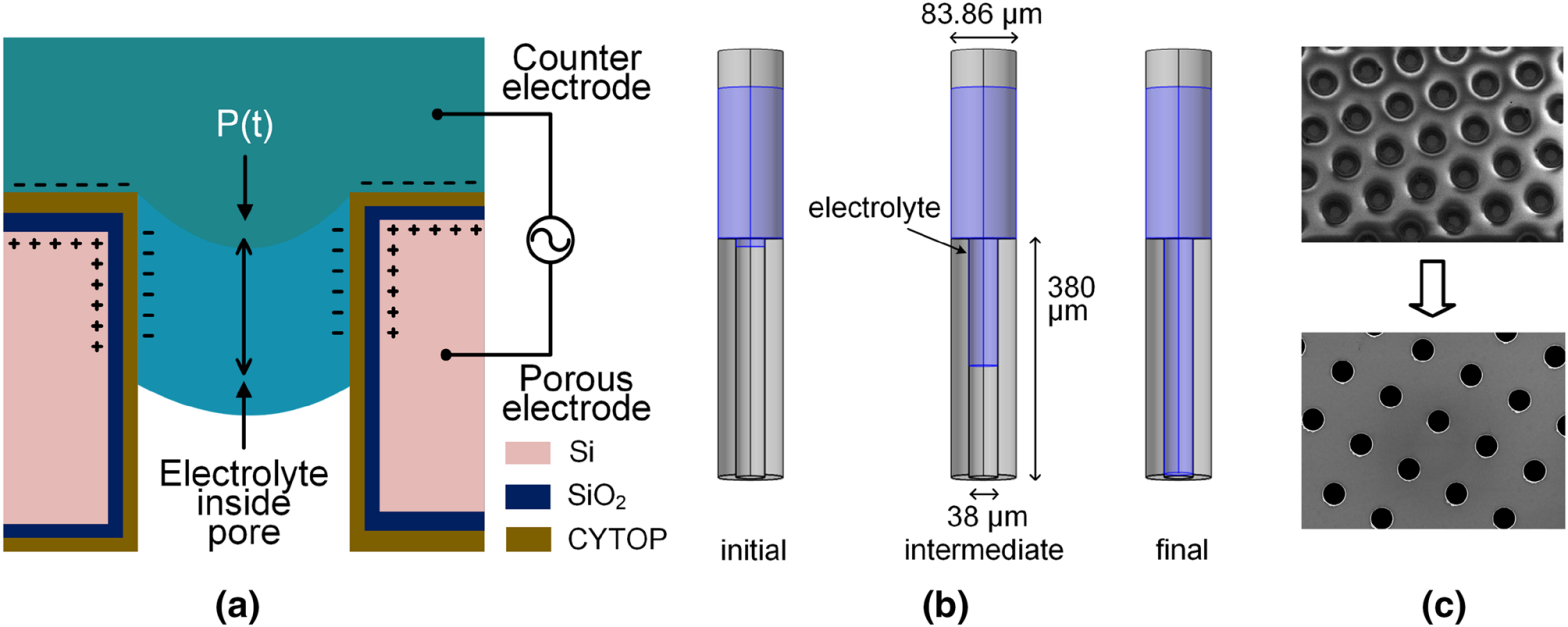
Overview of a high-surface area REWOD energy harvester, (**a**) working mechanism of REWOD with pulsating pressure *P*(*t*), (**b**) initial, intermediate, and final states of the electrolyte in a pore due to pulsating pressure, (**c**) etched pores using plasma deep reactive ion etching (DRIE).

## Materials and methods

High-surface area porous REWOD involves the process of transforming the kinetic energy from the mechanical modulation of liquid electrolyte into electrical energy. The change in the contact surface area within the porous electrode results in the generation of electrical power. Figure 1 shows the overview of the porous-REWOD energy harvester. Figure 1a shows the working mechanism and different layers of the porous REWOD electrode. The pulsating pressure *P*(*t*) causes the electrolytes to modulate inside the pores. Figure 1b demonstrates the three states of the electrolyte and the dimensions of the pore, while Fig. 1c shows the SEM images of the etched pores. The details of the fabrication and working mechanism are explained in the later sections. A more detailed description of the fabrication process is presented in our prior work^29^.

### Working mechanism

The working mechanism of the porous-REWOD sensor can be explained as the change in the EDL capacitance during the mechanical modulation. A high surface area electrode having uniform pores provides a much higher surface area compared to the planar area, thereby significantly increasing the electrode–electrolyte interfacial area during modulation. EDL can be significantly enhanced by increasing the electrode–electrolyte interfacial area. Under applied pulsating pressure *P*(*t*), the liquid electrolyte is inserted and retracted in and out of the pores at a given oscillation frequency as shown in Fig. 1a, generating alternating current as a direct result of periodically changing capacitance. *P*(*t*) is applied to the pores by compressing the air inside a syringe that is connected to a custom mechanical oscillator. In addition to the porous electrode, the housing that contains the electrolyte acts as the second electrode. The applied oscillating pressure results in periodic electrolyte modulation. Figure 1b illustrates the three states of the electrolyte in the pores: the electrolyte is just getting inside the pore in the initial state, in the intermediate state, the electrolyte is halfway through the pore depth, and the electrolyte is completely filling the pore in the final state. The modulation process goes on due to the periodic application and retraction of the pulsating pressure. The total time period for filing the pore completely depends on the time-dependent pressure *P*(*t*) according to the Washburn equation^38,39^. The applied pressure to the micro-pores ranges between 1.5–16 kPa, which is in the range of Laplace capillary pressure and can be expressed as:

1 $$\begin{aligned} P = 2 \gamma cos\theta / r \end{aligned}$$

where *P* is the amount of the required pressure for the liquid to fill into the micropores, $\gamma$ and $\theta$ are the surface tension between the liquid and the air, and the contact angle between the electrolyte and CYTOP coating, as shown in Fig. 1a. As soon as the applied pulsating pressure reaches the Laplace capillary pressure, the electrolyte starts penetrating into the pores. This modulation results in the periodic change in the electrode–electrolyte contact surface area, thus producing surface charge density. The electrical energy is then generated from the change in the surface charge density.

### Device fabrication

A very low resistance (0.001–0.005 $\Omega$-cm), 100 mm diameter, and 380 $\upmu$m thick double-sided polished silicon wafer (University Wafer) is used to fabricate the porous electrodes. Two uniform diameters of 38 $\upmu$m and 100 $\upmu$m pores within a circular area of 3.14 $\hbox {cm}^2$ (1 cm radius) are fabricated for two different samples. Within the given area, the number of 38 $\upmu$m pores is 51,877 and that of 100 $\upmu$m pores is 4681, thereby enhancing the total available surface area by 8.3 and 2.66 times, respectively, compared to the planar surfaces. A summary of the pore geometry and total surface area is presented in Table 1. The total surface area increases to 26, and 8.4 $\hbox {cm}^2$, respectively, in which most of the portion is coming from the wall surface of the pores.

**Table 1:** Pore geometry and total surface area of REWOD electrode.

Pore diameter ($\upmu$m)	Number of pores	Planar surface area (cm$^2$)	Porous surface area (cm$^2$)	Total surface area ($cm^2$)
38	51,877	2.553	23.53	26.09
100	4681	2.774	5.590	8.36

A pore pattern is created using the AutoCAD DXF file to make a high-resolution chrome mask in order to fabricate the porous REWOD device. KL6008 positive photoresist (Kemlab Inc.) was spin-coated on the wafer at 300 rpm for 5 seconds (spread cycle), and 600 rpm for 45 seconds (spin cycle). This process provides the desired photoresist thickness of $\sim$ 10–12 $\upmu$m as required to protect certain areas of the wafer during deep reactive ion etching (DRIE) later in the fabrication process. The wafer with a photoresist was cured for 150 s at 105 $^{\circ }$C on a hot plate. The cured wafer was then exposed under UV light for 45 s at 210 mJ/cm$^2$ of exposure broadband. Subsequently, the UV exposed wafer was immediately developed using 0.26N TMAH developer for $\sim$2 minutes, rinsed with deionized water, and nitrogen air-dried. The pore patterns and the photoresist thickness on the developed wafer were verified using Alpha-Step D-300 Stylus Profiler (KLA Corporation). The patterned wafer was etched to create through-pores using DRIE (Oxford 100 ICP) and deposited with $\hbox {SiO}_2$ dielectric using plasma CVD (Oxford Plasmalab 80), as shown in Fig. 1c. The anisotropic plasma DRIE was performed in the presence of Sulfur Hexafluoride ($\hbox {SF}_6$) at a flow rate of 80 sccm and Octafluorocyclobutane ($\hbox {C}_4$ $\hbox {F}_8$) at a flow rate of 90 sccm under a vacuum pressure of 7.5 $\upmu$torr. Once etched, the wafers were thoroughly cleaned before CVD deposition of $\hbox {SiO}_2$. After $\hbox {SiO}_2$ deposition, the wafer was deposited with an additional layer of hydrophobic material. A fluoropolymer, CYTOP (CTL-809M), and its solvent (CT-Solv. 180), both purchased from AGC Chemicals Company, were mixed together at a 1:5 ratio by weight. The porous wafer was dip-coated in the fluoropolymer solution. The drying process of the dip-coated wafers included 10 s of the vertical drain, 5 minutes of soft dry at 80 $^{\circ }$C, and a final dry for 12 minutes at 200 $^{\circ }$C for complete evaporation of the solvent. Both of the drying steps were performed with the wafer suspended 2 inches over the hot plate to prevent the clogging of the pores. Since the entire wafer was coated with $\hbox {SiO}_2$ during the CVD, and with CYTOP during the dip-coating process, a small portion of the wafer was treated with hydrofluoric acid (HF) to remove the $\hbox {SiO}_2$ and the CYTOP coating for the electrical conduction to a wire lead. HF etching of the conducting area is different from the fabrication of creating pores. The sample is held with a tweezer and a small portion away from the patterned pores is dipped in the HF solution for $\sim$ 4–5 minutes. The process is carried away until the CYTOP/$\hbox {SiO}_2$ layers are completely removed from that area. This portion of the sample is used for conduction during the current generation.

## Modeling of REWOD

In this section, the electrical equivalent circuit modeling of the porous-REWOD is presented. An analytical model is developed considering the significant parameters such as the dielectric material properties, pore variables (diameter, depth, depth-to-diameter aspect ratio), charge density of the surface, frequency of modulation, and externally applied mechanical pressure, etc. The analytical model is implemented in MATLAB, while the electrostatic physics-based model is implemented in COMSOL.

### Analytical modeling

In the porous-REWOD system, the electrolyte is forced in and out of pores under the influence of an oscillating pressure, which is described in the previous section. The pressure is set to oscillate at the range of 1–5 Hz, which is also the frequency range of normal human movement activities (for example walking, running, jogging, etc.). The surface area consists of two parts: the constant area, which is a non-porous, planar area between the pores that touches the electrolyte constantly, and a variable area, which is the area of the pore walls that contact with the electrolyte as it is inserted/retracted into the pores. The surface area of a single pore can be considered as the surface area of a cylinder, and the height of the cylinder changes as the penetration depth of the electrolyte changes during the modulation. Due to the change in the electrode–electrolyte interfacial area, the capacitance also varies proportionally. In this work, two different porous electrodes are considered, which have two different pore diameters (38 and 100 $\upmu$m). Taking all the parameters such as the number of pores, dielectric thickness, dielectric constants, etc. into consideration, an equation-based model is developed and implemented in MATLAB. The model provides an estimation of the generated capacitance, voltage, current, and power density from the REWOD sensor.

**Figure 2:**
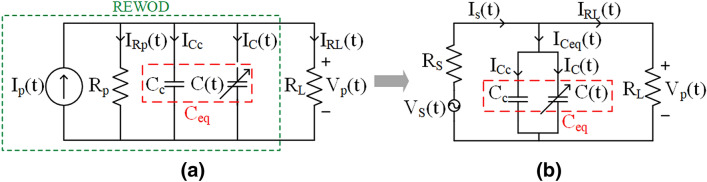
(**a**) Current-based, (**b**) voltage-based lumped circuit model for porous REWOD design.

A current-based electrical lumped circuit model of the porous REWOD sensor is shown in Fig. 2a. REWOD can be modeled as the current source ($I_p(t)$) in parallel with the equivalent impedance ($I_p(t) \parallel R_p \parallel C_{eq}$). $I_p(t)$ can be expressed as the current through the lumped elements (Fig. 2a).

2 $$\begin{aligned} \begin{aligned} I_p(t)&= I_{Rp}(t) + I_{Ceq}(t) + I_{RL}(t)\\ I_{Ceq}(t)&= I_{Cc} + I_{C}(t) \end{aligned} \end{aligned}$$

where $R_p$ is the parallel resistance between the electrodes and electrolyte, while the parallel capacitance is divided into two parts. $C_c$ is the constant capacitance that results due to the constant electrode–electrolyte inter-facial area in the planar portion of the electrode. *C*(*t*) is the time variant capacitance when the electrodes are oscillating, causing the electrolyte to go back and forth inside the pores. $R_L$ is the load resistance, which in this work is 11 k$\Omega$. $R_L$ is calculated from the current and voltage requirement of the wireless read-out circuitry. The total capacitance, $C_{eq}(t)$ of an individual pore can be calculated as:

3 $$\begin{aligned} C_{eq}(t)&= C_c + nC(t) = \frac{\varepsilon _0 \varepsilon _{eff}}{d_1 + d_2}(A_c+nA(t)) \end{aligned}$$

4 $$\begin{aligned} \varepsilon _{eff}&= \frac{d_1 + d_2}{\frac{d_1}{\varepsilon _{r1}}+\frac{d_2}{\varepsilon _{r2}}} \end{aligned}$$

where $\varepsilon _{eff}$ is the effective dielectric constant of the electrodes, and can be calculated from Eq. (4)^26^. *n* is the number of pores in the bottom electrode. $d_1$ and $d_2$ are the thicknesses of the hydrophobic layer (CYTOP) and the dielectric layer ($\hbox {SiO}_2$), respectively. $A_c$ is the constant electrode–electrolyte interfacial area, and *A*(*t*) is the varying contact area between the electrolyte and the electrode pores during the modulation. Since the pores on the electrode are considered as cylindrical, *A*(*t*) can be calculated as:

5 $$\begin{aligned} A(t)=2\pi rh(t) \end{aligned}$$

where *r* and *h*(*t*) are the radius and the varying electrolyte depth inside the pore, respectively. Due to the mechanical pressure, the depth covered in the cylindrical hole varies and so does the volume. The volume covered by the electrolyte inside the pores can be calculated as:

6 $$\begin{aligned} \begin{aligned} V_c(t) = \pi r^2 h(t) \\ V_c(t) = \frac{1}{2} r A(t) \end{aligned} \end{aligned}$$

From Eq. (6), it can be derived as:

7 $$\begin{aligned} \frac{dA(t)}{dt}=2\pi r\frac{dh(t)}{dt} \end{aligned}$$

According to the measurements, the total height of the pore is 380 $\upmu$m, so the liquid travels the distance of 0–380 $\upmu$m, and 2*r* is 38 $\upmu$m and 100 $\upmu$m for the two different samples used in the experiment. From Eqs. (3) and (7), we can develop the rate of change of capacitance ($dC_{eq}(t)/dt$).

8 $$\begin{aligned} \frac{dC_{eq}(t)}{dt}&= \frac{\varepsilon _0\varepsilon _{eff}}{d_1+d_2}\frac{dA(t)}{dt} \end{aligned}$$

9 $$\begin{aligned} \frac{dC_{eq}(t)}{dt}=\frac{dC_c}{dt}+n\frac{dC(t)}{dt}=0+n\frac{dC(t)}{dt}  \end{aligned}$$

10 $$ \frac{dC_{eq}(t)}{dt}=\frac{(2\pi rn)\varepsilon _0\varepsilon _{eff}}{d_1+d_2}\frac{dh(t)}{dt}$$

$dC_{eq}(t)/dt$ is used to evaluate the generated current and voltage for the porous REWOD. From Eq. (10), the rate of change of capacitor can be derived. Figure 2b shows the voltage-based lumped circuit model. The generated current $I_p(t)$ is converted into the voltage source $V_s(t)$ with a series resistance, $R_s$, using source transformation. The source voltage has two components: the DC bias voltage component, $V_{s,dc}$ and the AC component, $V_{s,ac}(t)$.

11 $$\begin{aligned} V_s(t) = V_{s,dc} + V_{s,ac}(t) \end{aligned}$$

The total current $I_s(t)$ flows through $C_{eq}(t)$ and $R_L$ ($I_{Ceq}(t)$ and $I_{RL}(t)$, respectively). Current through the equivalent capacitor can be found from the following equation.

12 $$\begin{aligned} I_{Ceq}(t) = V_{s,dc}\frac{\partial C_{eq}}{\partial t} + C_{eq}(t) \frac{\partial V_{s,ac}(t)}{\partial t} \end{aligned}$$

From Fig. 2a, we can write the voltages across the lumped elements as below:

13 $$\begin{aligned} \begin{aligned} V_p(t)&= R_p *I_{Rp}(t) = X_{Ceq}(t) *I_{Ceq}(t) = R_L *I_{RL}(t)\\ V_p(t)&= (I_{Rp}(t) + I_{Ceq}(t) + I_{RL(t)}) *Z_{eq}(t) \end{aligned} \end{aligned}$$

where $X_{Ceq}(t)$ and $Z_{eq}(t)$ are calculated as:

14 $$\begin{aligned} \begin{aligned} X_{Ceq}(t)&= \frac{1}{2 \pi f (C_c \parallel C(t))}\\ Z_{eq}(t)&= R_p \parallel X_{Ceq}(t) \parallel R_L \end{aligned} \end{aligned}$$

Thus, from Eq. (13), we can derive the following:

15 $$\begin{aligned} V_p(t) = I_{Ceq}(t) \left( \frac{X_{Ceq}(t)}{R_p} + 1 + \frac{X_{Ceq}(t)}{R_L}\right) *Z_{eq}(t) \end{aligned}$$

The total generated voltage thus can be calculated as:

16 $$\begin{aligned} V_p(t) = \left( V_{s,dc}\frac{\partial C_{eq}}{\partial t} + C_{eq}(t) \frac{\partial V_{s,ac}}{\partial t}\right) *\left( \frac{X_{Ceq}(t)}{R_p} + 1 + \frac{X_{Ceq}(t)}{R_L}\right) *Z_{eq}(t) \end{aligned}$$

Equations (2), (9), and (15) define the porous-REWOD’s current, rate of change of capacitance, and voltage, respectively. The model is implemented for the realistic prediction of the output currents and voltages from the porous REWOD transducer.

### COMSOL modeling

One of the main physics behind the REWOD phenomenon is the electrostatic energy stored in the capacitors formed in the system. This system is similar to a parallel-plate capacitor and can be modeled using COMSOL Multiphysics under Electrostatic physics of the AC–DC model. Using COMSOL Multiphysics, the total electrical charge on the surface and the capacitance is measured. In this work, the porous REWOD is modeled with the pore diameter of 38 $\upmu$m and simulated to obtain the capacitance and charge of the single pore, and then multiplied with the total pore numbers of the sample.

**Table 2:** Simulated model parameters.

Simulation parameters	Values ($\upmu$m)
Si cylinder diameter	83.86
Pore diameter	38
Pore depth	380
Dielectric hollow cylinder thickness	0.2
Empty pore diameter	37.8
Electrolyte diameter	37.8
Electrolyte height, $h_{com}$	10–370

**Figure 3:**
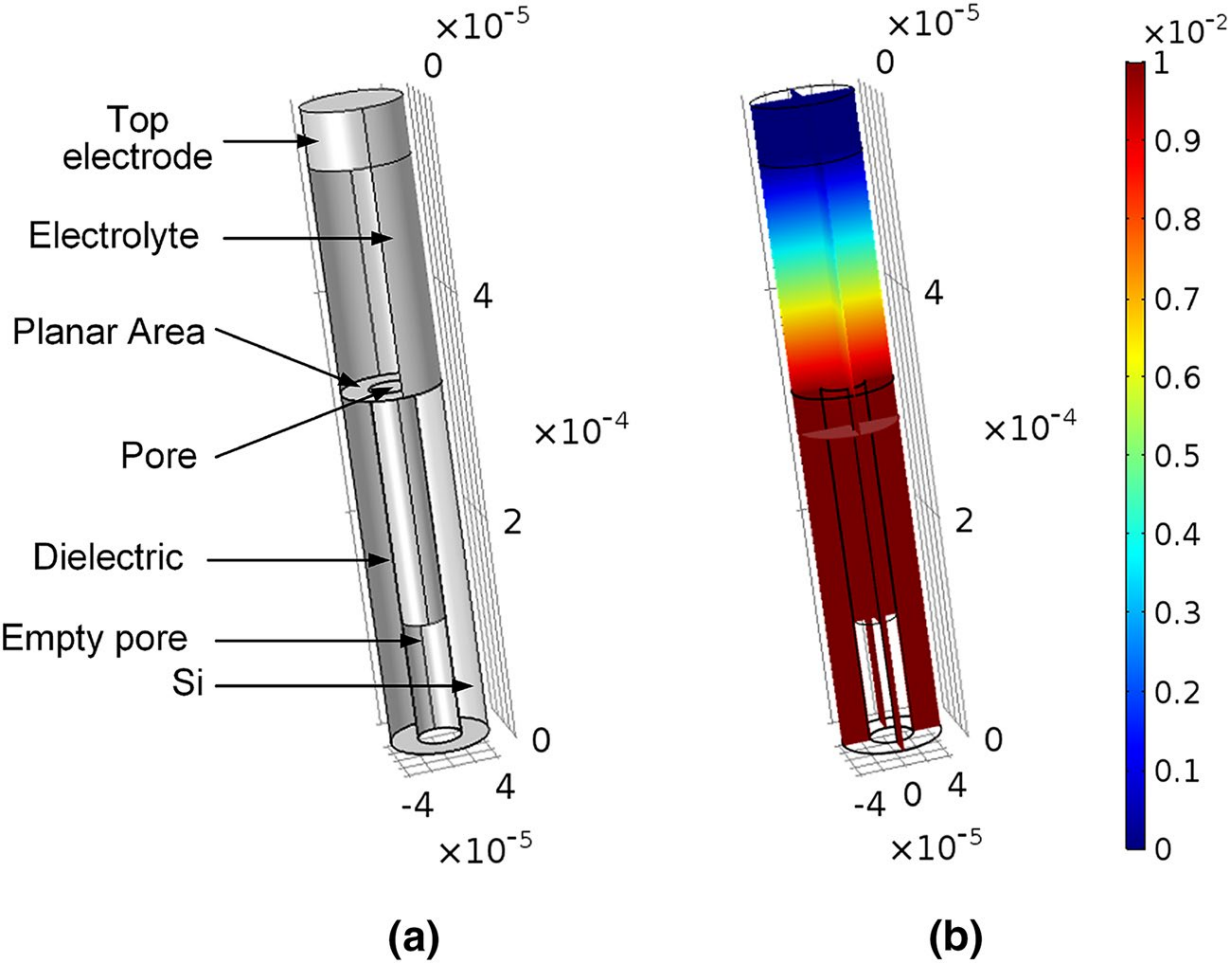
(**a**) 3D COMSOL model of a 38 $\upmu$m single pore, and (**b**) voltage distribution of the single pore.

The single pore is modeled by creating a hollow Silicon cylinder of 41.93 $\upmu$m outer radius, 19 $\upmu$m inner radius, and 380 $\upmu$m depth. The top surface of the hollow cylinder has an area of 4389.19 $\upmu$ $\hbox {m}^2$, which can be considered as a ring-shaped surface representing the planar surface area of a single pore. A hollowed cylinder of 200 nm thickness is modeled inside the pore to represent the dielectric layer. The dielectric layer represents the combined $\hbox {SiO}_2$ and CYTOP layers with the effective dielectric constant of 4.9. A 37.8 $\upmu$m diameter material is modeled inside the pore representing the electrolyte inside the pore. For a realistic simulation the height of the electrolyte layer, $h_{com}$ (Fig. 1b) is varied from 10 to 370 $\upmu$m to determine the current, voltage, capacitance, etc. for the various stages of the electrolyte going in and out of the pores. An electrolyte layer is also modeled on top of the pore followed by a pure conductive electrode layer. The dielectric constant of the electrolyte is assigned as 78^40^. The simulation parameters are summarized in Table 2. The 3D COMSOL model and the voltage distribution of the individual pore are shown in Fig. 3a,b, respectively.

‘Ground’ boundary condition is applied to all of the faces of the electrode and electrolyte and ‘Terminal’ boundary condition is applied to the faces of the silicon. Simulation is performed to obtain the capacitance ($C_{com}$) and the charge ($Q_{com}$) of the single pore. The simulation is performed multiple times by varying the height of the electrolyte layer inside the pore which represents the different stages of the pore being filled up and getting empty. This process is represented in Fig. 1b.

As the filling up of the pores depends on the applied pressure as well as the modulation frequency ($f_m$), $C_{com}$ and $Q_{com}$ are plotted against time. At time $t=0$, the pore is empty, at $t=T/2$ (where modulation period $T= 1/f_m$), the pore is completely filled, and again at $t=T$, the pore is completely empty, and the cycle continues. For each cycle the single modulation period, *T* is divided into 50-time increments. The electrolyte height at each time increment of the divided modulation time is calculated using Eq. (17) [for $t=0$ to $t=T/2$] and Eq. (18) [for $t=T/2$ to $t=T$].

17 $$\begin{aligned} h_{com}= & {} 10 + \frac{T}{50} * 360 \end{aligned}$$

18 $$\begin{aligned} h_{com}= & {} 370 - \frac{T}{50} * 360 \end{aligned}$$

A static simulation is performed for each time period with the height of the electrolyte placed accordingly. From the simulations, the capacitance $C_{com}$ is calculated at each time period. The total capacitance ($C_{tot}$) is calculated using Eq. (19), and the current ($I_{com}(t)$) is calculated using Eq. (20), by multiplying the total number of pores with the single pore capacitance and current, respectively.

19 $$\begin{aligned} C_{tot}&= n \times C_{com} \end{aligned}$$

20 $$\begin{aligned} I_{com}(t)&=n \frac{dQ_{com}}{dt} \end{aligned}$$

where *n* is the total number of pores in the porous REWOD. For the 38 $\upmu$m porous electrode, *n* = 51,877. This model is implemented in COMSOL and the evaluated results are shown in the later sections. The 100 um pore diameter modeling can also be done on COMSOL in a similar fashion.

## Energy harvesting circuitry

The energy harvesting circuit includes a bridge rectifier to convert the generated low-frequency (<10 Hz) AC signal to a DC value. In order to supply the 3.3 V to the motion detection circuit, a DC–DC boost converter is implemented to achieve a high power conversion efficiency followed by the rectifier. The detailed design is described in the following subsections.

### Bridge rectifier

**Figure 4:**
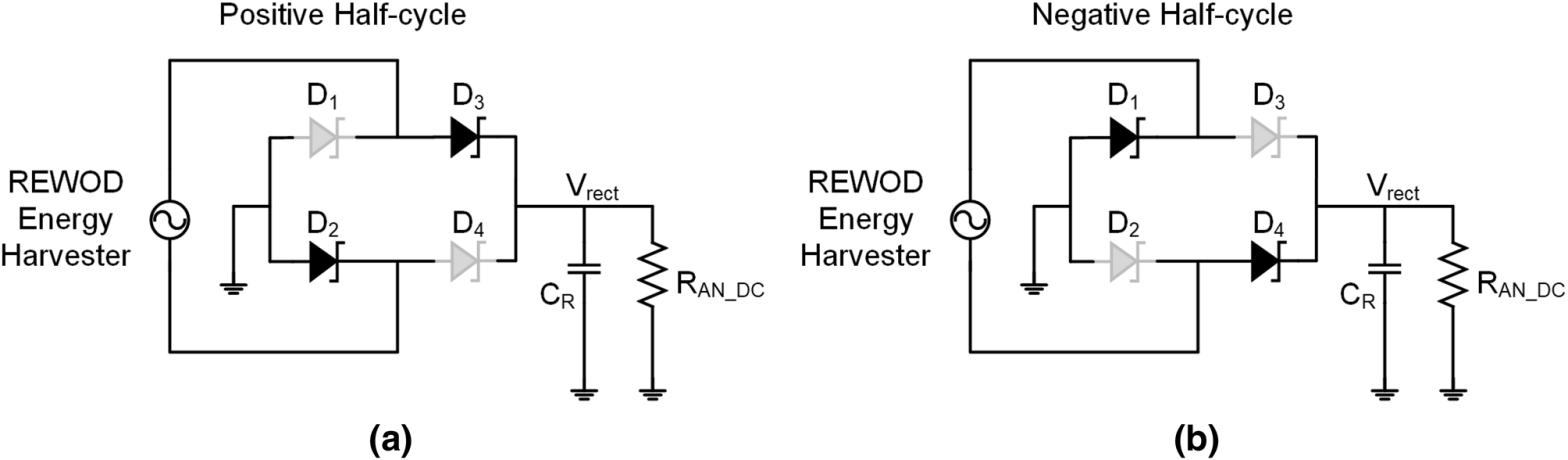
Schematic of the Schottky diode-based bridge rectifier, (**a**) working principle of the proposed rectifier during positive half-cycle, and (**b**) working principle of the proposed rectifier during negative half-cycle.

The proposed rectifier circuit is a bridge rectifier architecture that uses a commercially available PMEG2020AEA Schottky diode as shown in Fig. 4. The proposed Schottky diode has a minimum forward voltage requirement of 450 mV, and a maximum forward current of 2 A. During the positive half-cycle of the harvested AC signal, the diodes, $D_3$, and $D_2$ are forward biased, and the diodes $D_1$ and $D_4$ are reverse biased. Thus, the current flows from the positive node through $D_3$ and returns to the negative node through $D_2$ charging the capacitor, $C_R$ as shown in Fig. 4a. On the other hand, during the negative half-cycle of the harvested AC signal, the diodes, $D_1$, and $D_4$ are forward biased, and the diodes $D_3$ and $D_2$ are reverse biased as shown in Fig. 4b. Hence, the current flows from the negative node through $D_4$ and returns to the positive through $D_1$. During the negative half-cycle, the capacitor, $C_R$ discharges through the load resistor, $R_{AN-DC}$. The value of the capacitor, $C_R$ is set to be 10 mF to remove the output ripples within the frequency range of 1–10 Hz.

### DC–DC boost converter

**Figure 5:**
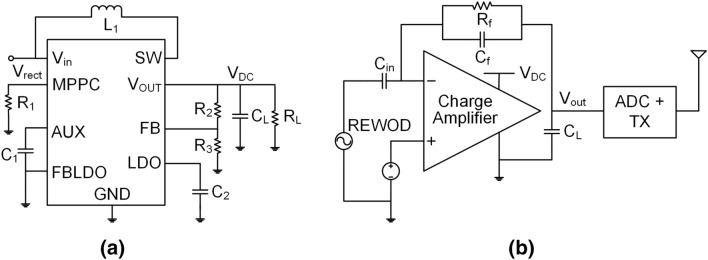
(**a**) Schematic of the LTC3105 DC–DC converter, (**b**) schematic of the wireless motion-detecting circuitry.

To make the system fully self-powered, the analog circuitry needs to be powered from the harvested energy. To provide the required 3.3 V supply voltage to the analog amplifier, a commercial DC–DC converter is used after the bridge rectifier that can provide a constant 3.3 V. The proposed DC–DC converter is a high-efficiency step-up DC–DC converter (LTC3105) by Linear Technology, which has a start-up voltage of 250 mV. The input voltage range of the proposed DC–DC converter varies from 225 mV to 5 V. The LTC3105 DC–DC converter is a 10-lead 3 mm $\times$ 3 mm DFN package and the schematic of the converter is shown in Fig. 5a. The device is integrated with a maximum power point controller (MPPC) that enables the device to operate at low voltage. The value of $R_1$ enables the user to set the lower range of the input voltage. To set the lowest input value of 250 mV, the value of the $R_1$ is chosen to be 25 k$\Omega$. A feedback resistive divider consisting of $R_2$ and $R_3$ is used at the output node that helps to adjust the output voltage between 1.6 and 5.25 V. To get a 3.3 V at the output node, the calculated values for $R_2$ and $R_3$ are 1.02 M$\Omega$ and 200 k$\Omega$, respectively. A load capacitor, $C_L$ of 10 $\upmu$F is chosen to get rid of the output ripple. The output load resistance, $R_L$ is calculated from the voltage and current requirements of the analog front-end and wireless transmission system, which results in a value of 11 k$\Omega$.

## Motion detector and wireless transmission circuitry

Due to the small amount of charge generation from the low-frequency and low-displacement movement, the signal coming from the REWOD transducer is anticipated to have a low amplitude. Therefore, a signal conditioning circuit is implemented to detect and amplify the motion signal. The readout integrated circuit (ROIC) includes a charge amplifier, ADC, and wireless transmission of the motion data. The charge amplifier converts the generated charge to a proportional voltage. The gain ($>6$ dB), bandwidth (1–10 Hz), input-impedance ($<1$ M$\Omega$), and input common-mode range (0–3.3 V) of the amplifier are designed to meet the motion signal specifications. It is then transmitted to the remote receiver through the transceiver and antenna. A detailed description of the circuits is given in the following subsections.

### Charge amplifier

A charge amplifier works as an interface between the REWOD-generated charge over a period of time and the read-out circuitry. Since the generated charge from the REWOD typically lies in a low-range value (several nCs), implementing the charge amplifier allows an improved signal-to-noise ratio (SNR) by transducing the charge into an output voltage. The charge input to the amplifier, or the AC current, $I_{in}$ is generated over the time duration *t*. The output voltage, $V_{out}$ is produced proportionately by converting the generated charge, $Q_{in}$ through the negative feedback network, $C_f$ and $R_f$, as can be seen in Fig. 5b. The transfer function for $V_{out}$ over time *t* can be expressed as follows:

21 $$\begin{aligned} V_{out}(t)&=-\frac{Q_{in}}{C_f}\exp {(-t/\tau )} \end{aligned}$$

22 $$\begin{aligned} \tau&= (R_p \parallel R_{in}).(C_p \parallel C_{in}) \end{aligned}$$

The electrical time constant $\tau$ is calculated from the input impedance of the charge amplifier ($R_{in} \parallel C_{in}$) and the equivalent electrical circuit model of the REWOD sensor ($R_{p} \parallel C_{eq}$). $R_{p}$ and $C_{eq}$ are calculated from the analytical and COMSOL-modeled REWOD sensor. The input capacitance of the charge amplifier is required to be larger than the feedback network for the charges to flow through the lower impedance path to ensure an efficient charge conversion. It also compensates for any slight variation in REWOD impedance due to the temperature, experimental setup, and so on. The schematic of the charge amplifier with the feedback network is shown in Fig. 5b. The mid-band gain of the amplifier can be calculated as below:

23 $$\begin{aligned} A_M = \frac{C_{in}}{C_f} \end{aligned}$$

$C_{in}$ and $C_f$ are chosen to be 1.8 pF and 0.9 pF, respectively to set the gain as 2 V/V. $C_{in}$ eliminates the DC components from the input AC signal. The bandwidth of the charge amplifier is designed to detect human physical activities within the motion frequency (1–10 Hz). The lower cut-off frequency ($f_L = 1/(2\pi \times R_f C_f)$) is chosen as 1 Hz. As the motion frequency increases, the input voltage to the amplifier also increases, which results in a clipped-off amplifier output voltage. Thus, the gain is set to a value so that it can amplify a broader range of input motion signals. The input common-mode range from the REWOD fluctuates from 0.5 to 1.125 V, thus a gain of 6 dB is chosen to cover this range, thus ensuring linear operation of the system.

In this work, a commercial rail-to-rail operational amplifier ADA4691-2 (Analog Devices) is used as the charge amplifier. ADA4691-2 is a low-power and low-noise (16 nV/$\sqrt{Hz}$) 8-lead amplifier with a dimension of 2 mm $\times$ 2 mm in a LFCSP (Leadframe Chip Scale Package) package. It operates within the input voltage range of − 0.3 to +3.9 V, with a common-mode rejection ratio (CMRR) of 90 dB. The high CMRR rejects and filters out the common-mode noise at the input.

### Wireless transmission

After initialization of the charge amplifier, the output voltages are sampled and digitized using a wireless microcontroller (MCU), CC430F5137 (Texas Instruments). CC430F5137 integrates the MSP430 MCU with the sub-1 GHz transceiver IC CC1101. It comes with a 7 mm $\times$ 7 mm QFN package. A 12-bit built-in SAR-ADC in the MCU digitizes the output with the analog input voltage range of 0 to 3.3 V. The ADC uses the internal built-in reference voltage of 1.5 V for implementing the SAR logic block. The conversion time for a full cycle is 2.4 $\upmu$s with the conversion clock as 0.45 MHz, which is the minimum clock frequency for the ADC. After the digitization, the transceiver radio transmits the signal to the remote receiver. Between the two modes of CC430 radio operation (active and low-power mode), the software-selectable low-power mode of operation is implemented. In this work, 2-GFSK (Binary Gaussian Frequency Shift Keying Modulation) is used at a data rate of 250 kBaud data rate. In GFSK modulation, a Gaussian filter is implemented in order to limit the spectral bandwidth of the output signal and to make the pulses smooth. The intermediate operating frequency was 152 kHz while transmitting at the 868-MHz frequency band at the transmitted power of 0 dBm. The wireless transmission system covers a distance of up to 20 m.

## Experimental results

### Modeled REWOD sensor

**Figure 6:**
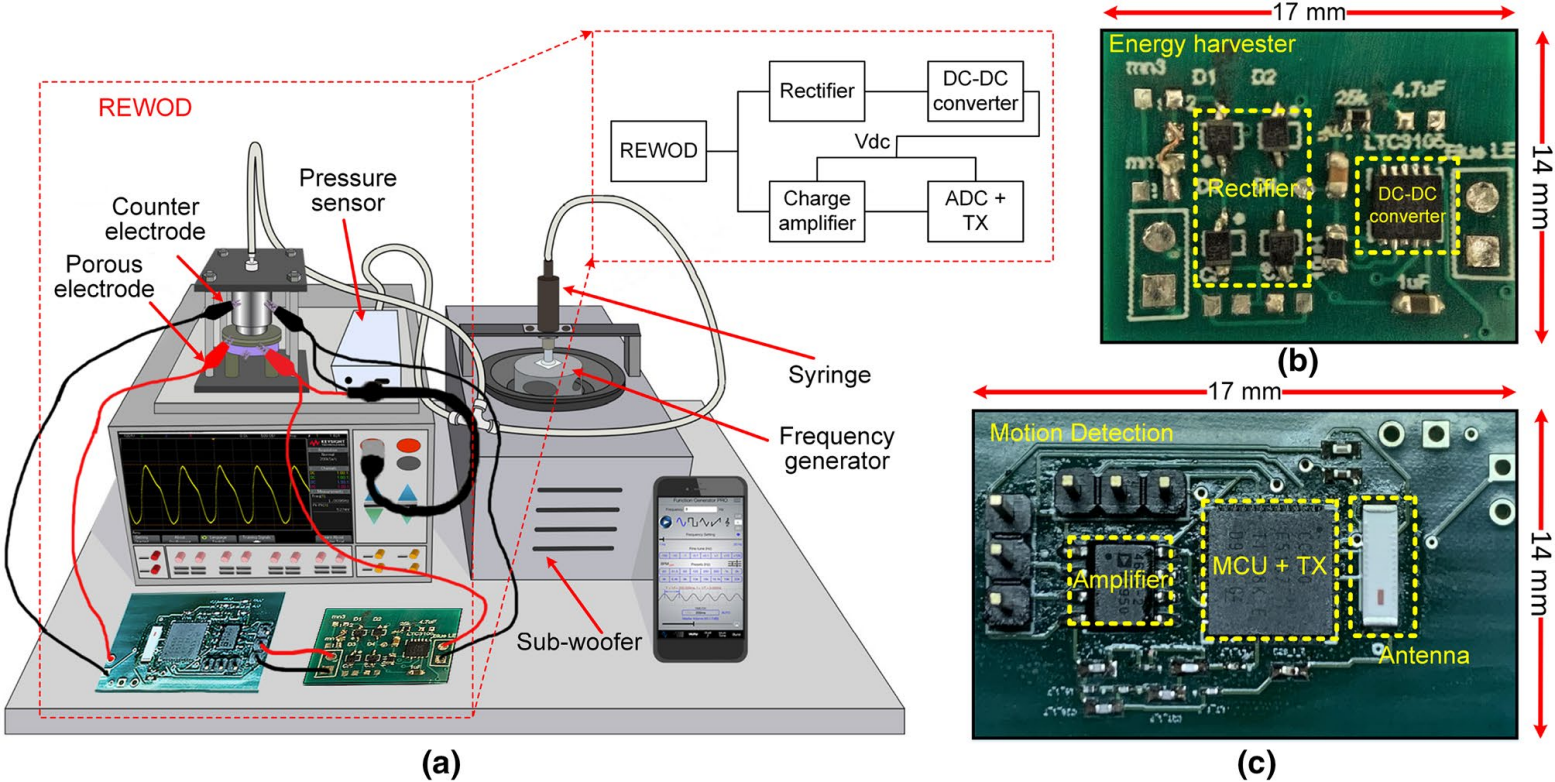
(**a**) Experimental setup for the REWOD energy harvester measurements, (**b**) fabricated PCB board of Energy harvesting circuitry, (**c**) fabricated PCB board of motion detecting circuitry.

**Figure 7:**
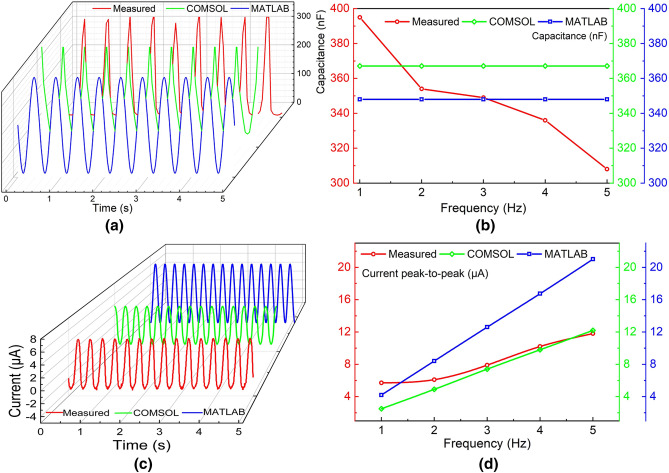
(**a**) Measured and modeled (COMSOL and MATLAB) representative capacitance over time at 2 Hz frequency of modulation for 38 $\upmu$m sample, (**b**) capacitance over the frequency of modulation, (**c**) measured and modeled (COMSOL and MATLAB) representative current over time at 2 Hz frequency of modulation for 38 $\upmu$m sample, (**d**) peak-to-peak current over the frequency of modulation.

Periodical change in the electrode–electrolyte interfacial area due to the externally applied mechanical force (the pulsating pressure) results in the generation of AC current. Prior to the experiments, several trial AC current measurements were performed by applying pulsating pressure in small increments starting with a lower limit ($\sim$1.5 kPa) to observe any increase in the magnitude of the AC current. The applied pressure should exceed the threshold value, otherwise, there would be insufficient pressure inhibiting the electrolyte from covering the entire pore depth. Excess pressure would cause the electrolyte leakage from the porous electrode to the bottom chamber of the test housing. Considering this issue, an optimum pressure is applied to the electrolyte. This is how optimum peak-to-peak pulsating pressures were determined for several modulation frequencies. A pressure sensor (PASCO PS-320) is used to measure the applied pressure within the frequency range of 1–5 Hz with a step size of 1 Hz. The physical set-up for measuring the REWOD energy harvester is shown in Fig. 6a. The porous electrode is considered as the working electrode, while another wire (also called a counter electrode) is connected to the upper aluminum housing that is in contact with the electrolyte. A syringe is vertically attached to a sub-woofer (8-inch 800 W Pyle, with a 400 W amplifier) for generating the pulsating pressure. The frequency and the amplitude of modulation are adjusted by a mobile phone application (Audio Function Generator PRO). Ample electrolyte is placed over the porous electrode to fully cover the surface area, also accounting for the entire pore volume of the pores.

Once the subwoofer-generated pulsating pressure exceeds the calculated optimum pressure, an EDL capacitor is formed between the electrode and the electrolyte. The porous-REWOD system is characterized by measuring the generated capacitance. An impedance and electrochemical measurement system AD5940 (Analog Devices) is used to measure the impedance (magnitude and phase angle) of the sensor. The equivalent impedance of the system is measured and compared with the modeled values. Figure 7a presents the measured, and both the COMSOL and the MATLAB-modeled capacitances at 2 Hz frequency for a representative sample of 38 $\upmu$m porous-REWOD. It can be seen from the figure that the capacitance values for all three are comparable, validating the model with the measurement. The maximum capacitance value goes up to 390 nF at 2 Hz for the measurement, while those of the modeled values are 369 nF and 348 nF. Figure 7b shows the maximum capacitance for the frequency range of 1–5 Hz. Although the measured values decrease with increasing frequency, both the modeled capacitance remains constant within the measured range. The mismatch may have been caused by the experimental setup of the penetrating electrolyte inside the pores.

In order to measure the output power density of the REWOD sensor, the generated current is measured using Keithley KickStart Software 2.0 (Tektronix) and 2401 Keithley Sourcemeter. Figure 7c presents the measured and both the COMSOL and the MATLAB modeled currents at 2 Hz of frequency for the 38 $\upmu$m porous sample. The measured current is 6.9 $\upmu A_{p-p}$, while the COMSOL and the MATLAB model show 4.9 and 8.4 $\upmu A_{p-p}$, respectively. The discrepancy between the measured and the modeled peak-to-peak current value in Fig. 7d may have resulted from not having the exact value of the pore diameter and the accurate displacement of the electrolyte in the fabricated device. The generated current from the REWOD energy harvester increases with the increase in the frequency of modulation. The maximum peak-to-peak current for the same sample is found as 11.83 $\upmu$A at 5 Hz modulation frequency. The total available planar surface area for a 1 cm-radius wafer is 3.14 $\hbox {cm}^2$, which results in a maximum current density of 3.77 $\upmu$A/$\hbox {cm}^2$. The lowest current density is generated from the 100 $\upmu$m diameter sample as 0.6 $\upmu$A/$\hbox {cm}^2$ at 1 Hz. Since introducing more pores to the same circular electrode area results in a higher surface area, it could eventually generate more current density at the expense of the robustness of the device.

### Bridge rectifier

**Figure 8:**
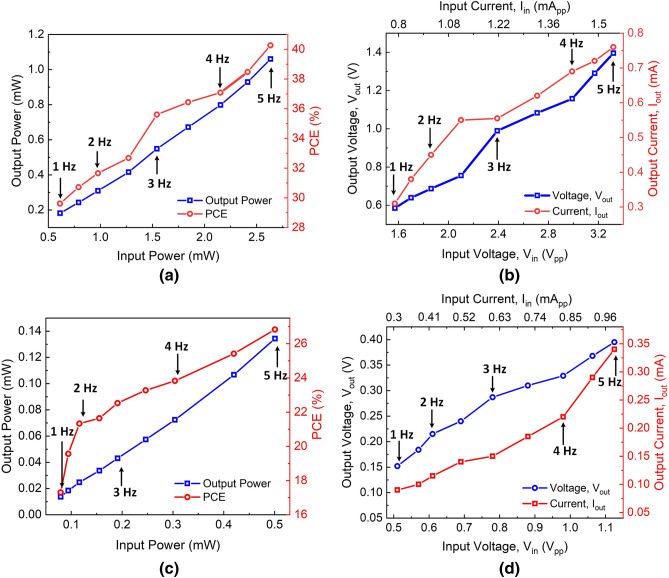
(**a**) Power output and power conversion efficiency, (**b**) output voltage and current for the energy harvested using 38 $\upmu$m porous diameter, (**c**) power output and power conversion efficiency, and (**d**) output voltage and current for the energy harvested using 100 $\upmu$m porous diameter.

**Figure 9:**
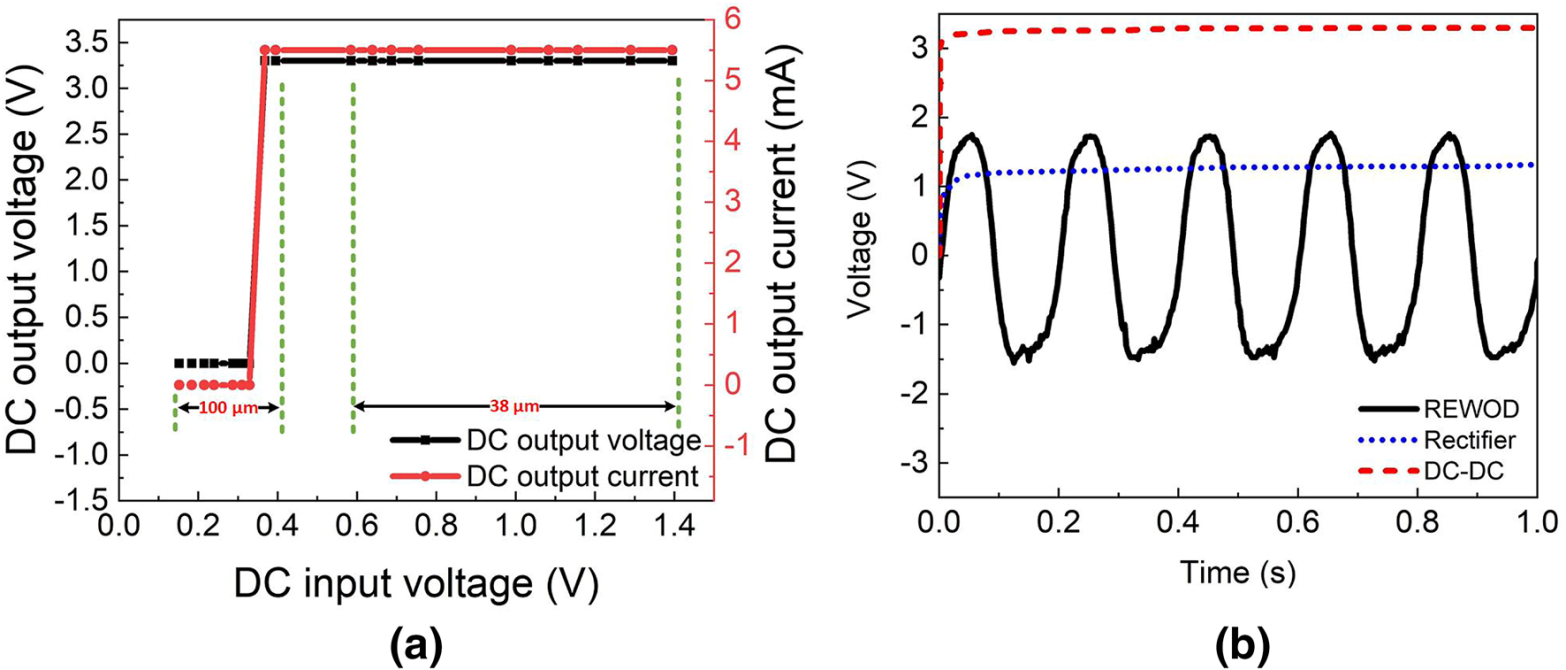
(**a**) Output voltage and current of the DC–DC converter for the harvested energy using 38 $\upmu$m and 100 $\upmu$m, (**b**) instantaneous voltage output from the REWOD, rectifier, and DC–DC converter.

The energy harvesting circuit is fabricated on a 14 mm $\times$ 17 mm printed circuit board as shown in Fig. 6b, which has the rectifier and the DC–DC converter. The rectifier and the DC–DC converter are individually characterized before experimenting with the REWOD system. The output power and power conversion efficiency (PCE) of the bridge rectifier for the frequency range of 1–5 Hz with a step size of 0.5 Hz is presented in Fig. 8a. The *x*-axes of the Fig. 8a,b represent the harvested power and voltage, respectively for the 38 $\upmu$m-diameter pore electrodes. The minimum power harvested from the REWOD system is 0.61 mW at 1 Hz frequency which gives 0.18 mW output DC power after the rectifier yielding 29.6% efficiency. The maximum PCE of the rectifier is achieved as 40.2% at 5 Hz frequency for an input power of 2.6 mW. Figure 8b shows the output voltage and current of the rectifier for the input voltage from the REWOD that corresponds to the input power of Fig. 8a. The output DC voltage ranges from 0.58–1.39 $\hbox {V}_{p-p}$ for the input voltage range of 1.57–3.32 $\hbox {V}_{p-p}$ for different frequencies. The maximum output current is measured to be 0.3 mA - 0.76 mA from the rectifier.

Figure 8c,d show the output measured data for the harvested signal when the pore diameter is increased to 100 $\upmu$m. The power harvested from the REWOD system varies from 0.08–0.5 mW and the corresponding voltage is between 0.51–1.12 $\hbox {V}_{p-p}$ for the varying frequency of 1–5 Hz. The maximum efficiency is measured to be 26.8% at 5 Hz frequency which provides 0.13 mW of output power and 0.39 V output voltage. The output voltage ranges from 0.15–0.39 $\hbox {V}_{p-p}$ providing an output power range of 0.09–0.34 mW for 100 $\upmu$m porous REWOD as shown in Fig. 8d.

### DC–DC boost converter

The measured voltage and current of the DC–DC converter are shown in Fig. 9a with respect to the output voltage from the REWOD system. The DC input voltage in Fig. 9a represents the rectified voltage for both the REWOD samples consisting of 38 $\upmu$m and 100 $\upmu$m pore samples. As shown in Fig. 9a, the DC–DC converter provides 3.3 V and 5.5 mA current for all the rectified voltages for 38 $\upmu$m pore diameter sample. However, for the 100 $\upmu$m pore diameter, the DC–DC converter is not able to provide a constant 3.3 V until the input voltage reaches 0.36 V. This is due to the fact that the current provided by the rectifier is not sufficient to turn on the DC–DC converter, which has a minimum current requirement of 0.28 mA. Figure 9b shows the rectified output with the instantaneous voltage from the REWOD at 5 Hz frequency. The rectified output takes a response time of 0.3 s to get to the saturated output voltage of 1.2 V at 1 Hz frequency, while at 5 Hz frequency, it takes 70 ms to reach to 1.2 V output value. The DC–DC converter up-converts the voltage to 3.3 V from the 1 V rectified DC voltage.

### Motion detecting circuitry with wireless data transmission capabilities

In the case of motion sensing and fall detection, a sensitive ROIC is crucial in terms of the transducer and charge amplifier voltage change over the low motion frequency. The REWOD transducer sensitivity is found to be 122.5 mV/Hz. The ROIC is designed to meet the sensitivity requirement of the transducer. Optimizing the power dissipation for the motion detecting circuit is also crucial for the motion sensor while ensuring stability and sensitivity. Filtering the raw motion data to eliminate the noise components is also significantly important because low-frequency motion can contribute to the noise figure. The ROIC is designed and characterized taking all these constraints into consideration. A band-pass filter is implemented with the charge amplifier to amplify the motion signal within 1–10 Hz frequency. The charge amplifier and the wireless transmission circuit are fabricated on a 17 mm $\times$ 14 mm PCB board, as shown in Fig. 6c.

**Table 3:** Measured input impedance of the charge amplifier.

Frequency (Hz)	$R_p (k\Omega )$	$C_p (nF)$	$Z_{eq} (k\Omega )$
1	383	321	216
2	196	159	140.8
3	187	94.2	120.4
4	181	78	92
5	173	59	74

In order to characterize the charge amplifier, the input impedance is measured and presented in Table 3. It shows the equivalent impedance decreases with the increase in the frequency of modulation. The input impedance ranges from 74 to 216 k$\Omega$ for the frequency range of 1–5 Hz, with the capacitance in the nF range. Since the feedback network has a much smaller impedance (few pFs), most of the current generated by the REWOD goes through the feedback network, ensuring efficient charge conversion.

**Figure 10:**
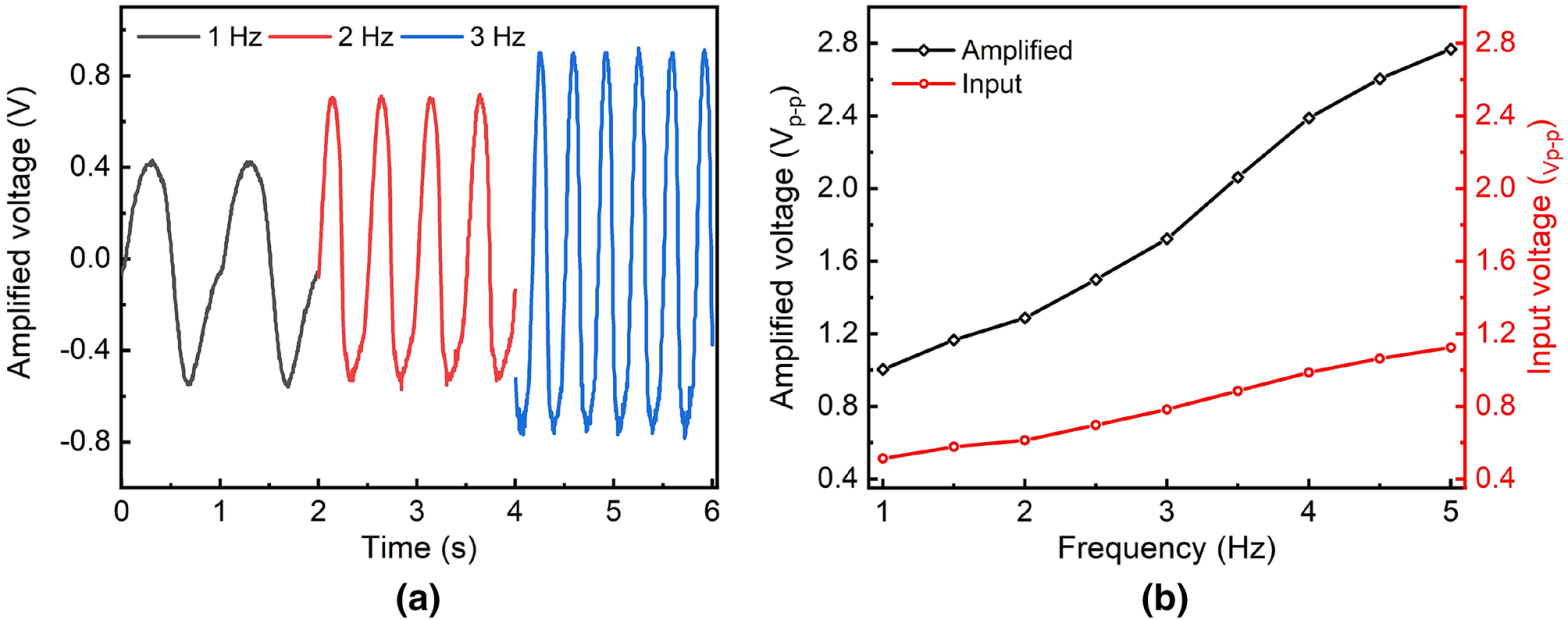
(**a**) Reconstructed amplified signal at the receiver for 1.0–3.0 Hz, (**b**) Amplified output of the charge amplifier and input voltage from the REWOD.

The output AC voltages are measured for a frequency range of 1–5 Hz with a 0.5 Hz step size. The motion-generated charge is translated into a proportional voltage through the charge amplifier. After the sampling and digitization, it is transmitted wirelessly to the remote receiver. An MSP430-CCRF (Olimex transceiver with microcontroller evaluation board) is used as the receiver. The power consumption of the recording circuit is 0.3 mW. The quiescent current consumption of the amplifier is found as 7 $\upmu$A at low-power mode, while that of the transceiver is 22 $\upmu$A. A LabVIEW GUI is developed to convert the digital data to the analog domain. A representative sample of measured AC voltage over time for 6 seconds for the 38 $\upmu$m pore size electrode at 1.0–3.0 Hz frequency is shown in Fig. 10a. It shows the reconstructed motion signal after the wireless transmission for a distance of 2 m. Figure 10b shows the peak-to-peak voltages for a 38 $\upmu$m diameter pore electrode. AC voltage increases with the increasing pulsating pressure (frequency), showing almost a linear relationship between the voltage and the modulation frequency. The amplified peak-to-peak voltage ranges from 1.0–2.76 V for the given frequency range, with the input range as 0.5–1.2 V. The average sensitivity of the charge amplifier is calculated as 353 mV/Hz over the frequency range of 1–5 Hz. The time interval between the two different motion data is calculated as 0.4 ms, which shows the readiness of the sensor triggered by any movement.

The total power consumption of the motion-detecting circuitry is measured as $\sim$ 1 mW. The power density from the REWOD energy harvester without any load is calculated as 0.43 $\upmu$W/$\hbox {cm}^2$, which can be increased by increasing the number of pores per area, thus utilizing the high surface area. The harvested power can be enhanced by using the COTS-based energy harvesting rectifier and DC–DC boost converter. The system is able to generate sufficient energy to power up the motion-detecting circuitry, making it suitable for a self-powered motion sensor. In future work, the device is planned to be mounted under the shoe sole, where human motion and weight would generate the pulsating pressure. The harvested electrical power from this pressure will provide the energy to the motion detecting circuit, thus making it a self-powered sensor.

## Conclusion

This paper presents a high surface area porous REWOD energy harvesting system without any external bias. The novelty of the work includes the modeling of the REWOD sensor and the low-power electronic interfaces. The generated capacitance and current density are modeled both analytically and using a multiphysics-based approach. While the modeling helps to learn about the generated total power density, the values are also comparable to the measured power. We have also developed a COTS-based electronic circuitry for the energy harvesting and signal conditioning system. The energy harvesting achieves an efficiency of 40.2% with the produced output voltage as 3.3 V. The wireless motion signal conditioning circuit also detects the low-frequency movements, which makes the system compatible with the motion/health monitoring system.
